# Folate transporters at the plasma membrane of fungi and plants: specificity, mechanism of transport, and importance

**DOI:** 10.1042/BST20260103

**Published:** 2026-10-05

**Authors:** Nikita Vashist, Anand Kumar Bachhawat

**Affiliations:** Department of Biological Sciences, Indian Institute of Science Education and Research, Mohali, SAS Nagar, Punjab 140306, India

**Keywords:** Alternating-access model, Folate, Glutathione, Oligopeptide transporter family, OPT1/HGT1, Transporter

## Abstract

Folates are essential metabolites of the cell and need to be acquired either by transport or biosynthesis. All metazoa including humans, some bacteria, and some protozoa acquire it solely through specific transporters. Plants and fungi, in contrast, have the capacity both for *de novo* biosynthesis and for uptake through plasma membrane folate transporters. These recently identified transporters belong to the oligopeptide transporter (OPT) family. Folate transporters from this family have been identified in yeasts and other fungi, including pathogenic fungi. They have also been observed in plants such as *Arabidopsis thaliana* and have the ability to transport both synthetic and natural folates including the naturally abundant 5-methyl tetrahydrofolate. In *Saccharomyces cerevisiae*, the folate transporter Opt1p (Hgt1p) is a proton-coupled transporter that was already known to transport glutathione (GSH). While in some organisms a single transporter is able to transport both substrates, in other organisms separate transporters in this family exist for folate and GSH. Mutational, kinetic, and functional studies of *S. cerevisiae* Opt1p have identified residues involved in substrate translocation, including those specific for folate or GSH recognition, as well as for proton-coupled transport. These studies suggest, in analogy to a human proton-coupled folate transporter and a plant OPT family phytosiderophore transporter, HvYS1, that proton-driven folate transport follows an alternating access mechanism with features described here. These transporters have implications for drug development strategies targeting the folate pathway of fungi and have importance in folate biofortification strategies for crops.

## Introduction

Folates, also known as vitamin B9, are essential metabolites in all living systems. They function primarily as carriers of one-carbon units in diverse metabolic reactions [1]. Folates play a critical role in many pathways, including in the synthesis of nucleotides and amino acids such as serine and glycine, and in the SAM-dependent methylation reactions crucial for epigenetic and other cellular functions [2].

Chemically, folates are composed of three distinct moieties: a pterin ring, *p*-aminobenzoic acid (PABA), and one or more glutamate residues. Folic acid is a synthetic form of folate where the pterin ring is fully oxidized. It has to be reduced to dihydrofolate (DHF) and then to tetrahydrofolate (THF) to become biologically active [2]. The naturally occurring folates are all derivatives of THF. These derivatives differ in the oxidation state of the pteridine ring, the type of one-carbon group attached at the N5 and/or N10 positions, and the length of the polyglutamate tail. Such structural variations determine the metabolic roles, stability, and intracellular distribution of individual folate species. The most abundant species is 5-methyl THF [3–5].

## Folate acquisition: biosynthesis and transport

Folates are not synthesized by all organisms. Only plants, yeasts and other fungi, some bacteria, and some protozoa have this capability. Metazoans, including insects, animals, and humans, do not synthesize folates but obtain them from their diet.

Folate biosynthetic pathways are evolutionarily conserved among organisms that synthesize folates, but there are differences in enzyme organization, multifunctionality, and intracellular localization, which vary across kingdoms [6,7]. Folates are synthesized from three precursors: a pteridine moiety derived from GTP, PABA from chorismate, and glutamate residues. Some bacteria lack the complete pathway and thus salvage intermediates; for example, many lactic acid bacteria require PABA supplementation [8]. In fungi, folate biosynthesis involves multifunctional enzymes and is partitioned between the mitochondria and cytosol [9–11]. In plants, folate biosynthesis shows greater compartmentalization and involves the cytosol, plastids, and mitochondria. The pteridine branch occurs in the cytosol; PABA synthesis in plastids; and both branches converge in mitochondria for DHF and THF formation [7].

In organisms that do not synthesize folates, these have to be acquired from the diet or environment. As folates are highly hydrophilic and predominantly exist as negatively charged anions, they cannot readily diffuse across the membrane lipid bilayers. Consequently, their transport across membranes depends on dedicated transport systems. Passive diffusion occurs only at extremely high pharmacological concentrations and is negligible under physiological conditions [12]. After entry into the cell, folates are polyglutamated, which restricts their efflux. The intracellular transport of folates or their catabolites between organelles therefore requires prior deglutamation to monoglutamated forms before they can be transported [2].

## Folate transporters are observed in different transporter families

Folate transport in different organisms and across different organelles is mediated by multiple transporter families that differ in structure, localization, and transport mechanism (Table 1).

**Table 1:** Summary of Folate transporters found in different organisms/organelles

Name	Family	Organisms/organelle	Mechanism	Reference
**SLC19A1 (RFC)**	Solute carrier (SLC19) family	Mammals/plasma membrane	Bidirectional anion exchange	[13]
**SLC46A1 (PCFT)**	Solute carrier (SLC46) family	Mammals/plasma membrane	Proton coupled	[14]
**FolT**	ATP binding cassette (ABC) transporters	Gram positive bacteria/plasma membrane	ATP hydrolysis	[15]
**MRP**	ATP binding cassette (ABC) transporters	Mammals/plasma membrane; plant/vacuolar membrane	ATP hydrolysis	[16,17]
**FBT**	Folate-biopterin transporter (FBT) family	Cyanobacteria/plasma membrane; Protozoan/plasma membrane; Plant/chloroplast membrane	Uniport/symport/antiport	[18,19,20]
**AtFOLT1**	Mitochondrial carrier family	Plant chloroplast membrane	Electrochemical gradient	[21]
**OPT**	Oligopeptide transporter family (OPT)	Yeasts, fungi, and plants/plasma membrane	Proton coupled	[22]

Abbreviations: RFC: reduced folate transporter, PCFT: proton-coupled folate transporter, MRP: multidrug resistance-associated proteins.

In addition to these transporters, in mammalian cells, folate can enter into the cell through folate receptors (FOLR1–3), which are high-affinity folate-binding proteins and mediate uptake through receptor-mediated endocytosis, which occurs by vesicular internalization and endosomal acidification, leading to release of folate into the cytosol [23,24].

A member of the SLC25 family in plants, SLC25A32, was initially proposed to act as a mitochondrial folate carrier localized to the inner mitochondrial membrane, mediating the transport of reduced folate monoglutamates [25,26]. However, studies with the mammalian SLC25A32 [27] suggest that it functions as a mitochondrial FAD transporter rather than a folate carrier, thus also calling into question the report with the plant homolog.

## The plasma membrane folate transporters of fungi and plants

Fungi, including yeast, and plants are capable of *de novo* folate biosynthesis. Consequently, it was long considered that these organisms lacked active folate transporters in their plasma membranes. However, accumulated physiological and genetic evidence suggests otherwise. For example, in *Saccharomyces cerevisiae*, disruption of folate biosynthetic genes resulted in folate auxotrophy, where the mutants could grow when supplied with external folates [28,29]; in plants, indirect evidence for plasma membrane folate transport came from observations that folate supplementation enhances resistance to biotic stress [30] and also significantly improves productivity in pea and barley [31].

Recent identification of plasma membrane folate transporters operating in these organisms has resolved the issue. Studies with the yeast *S. cerevisiae* revealed that a member of the oligopeptide transporter (OPT) family, a family completely unrelated to the hitherto known folate transporter families, was responsible for folate transport across the plasma membrane [22]. Even more surprising was that the folate transporter is the well-known high-affinity glutathione (GSH) transporter Opt1p [32]. Competition between GSH present in the yeast extract used to prepare YPD medium might explain why previous experimental efforts toward the identification of a folate transporter in *S. cerevisiae* were unsuccessful. The two substrates likely interfered with each other. Biochemical uptake experiments revealed that Opt1p could not only efficiently transport folinic acid (5-formyl THF) but also the physiologically more abundant 5-methyl-THF. The different substrates of Opt1p and their chemical structures are shown in Figure 1. Examining OPT family members in other organisms revealed many of them being capable of folate transport. Examining OPT family members from other organisms by heterologous expression in a *S. cerevisiae opt1Δ* strain revealed that many are capable of folate transport [22,33]. Table 2 provides the list of members that were evaluated for their role in folate or GSH uptake.

**Figure 1:**
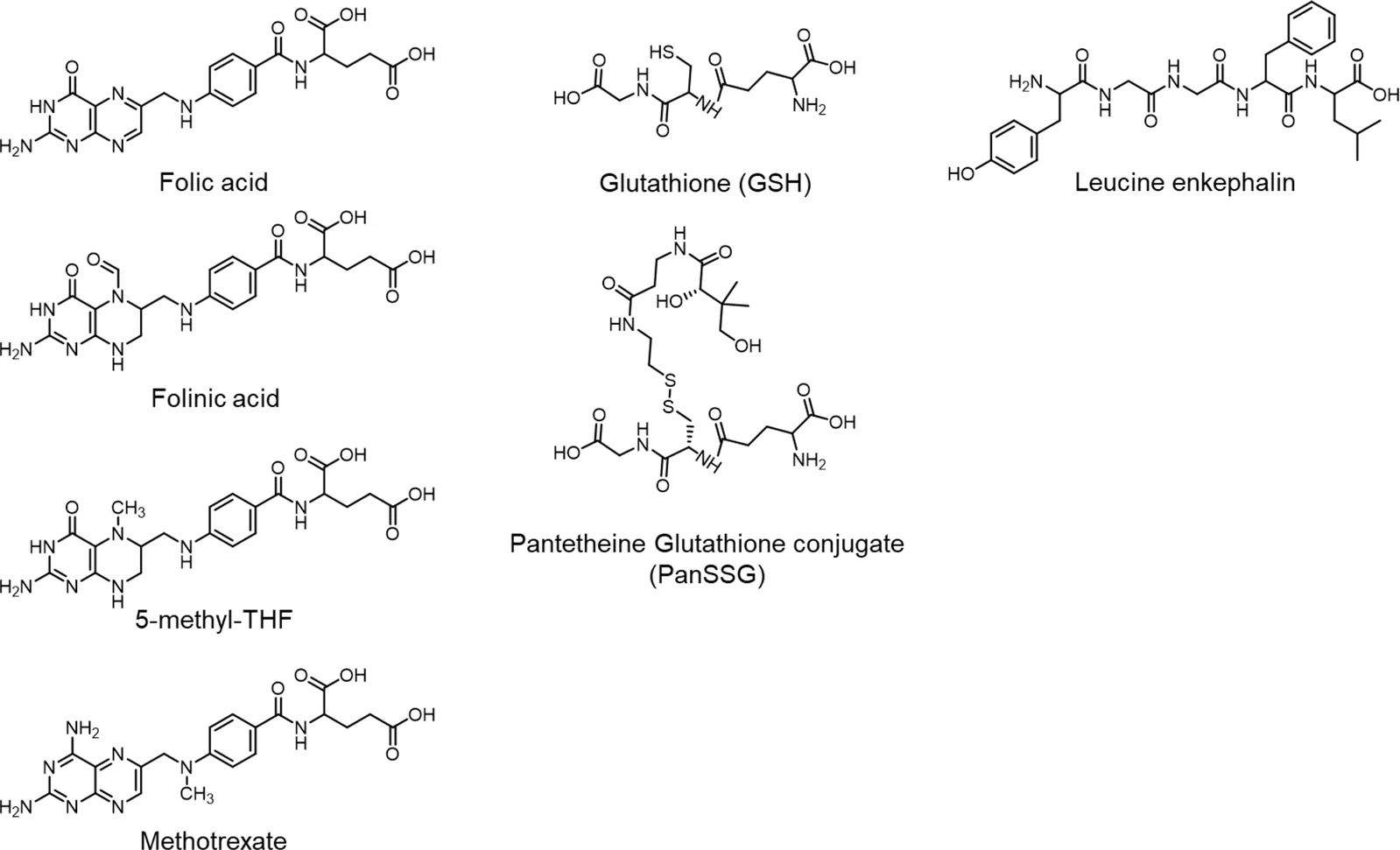
Substrates of Opt1p

**Table 2:** Different yeast and fungal folate transporters

Organism	OPT transporter	Role in folate uptake	Role in GSH uptake
***Saccharomyces cerevisiae***	ScOPT1	Yes	Yes
***Kluveromyces lactis***	KlOPT1	Yes	Yes
***Cryptococcus neoformans***	CnOPT1	No	Yes
***Candida albicans***	CaOPT1	Yes	No
	CaOPT7	No	Yes
***Apergillus fumigatus***	OPTB	Yes	No
	OPTH	Yes	No
***Arabidopsis thaliana***	AtOPT2	Yes	No
	AtOPT4	Yes	Yes
	AtOPT6	Yes	Yes

Interestingly, while some organisms (such as *S. cerevisiae* and *Kluveromyces lactis*) have transporters with the ability to transport both folates and GSH, others such as *Candida albicans* have transporters with clearly segregated functions: CaOpt1 transports folate but not GSH, and CaOpt7 transports GSH but not folate [22,34]. All 9 OPTs of *Aspergillus fumigatus* were examined, and only two could transport folate [22].

Since folate biosynthesis occurs in fungi but not in humans, this pathway has been proposed as an attractive antifungal target [35,36]. However, the presence of dedicated folate transporters in fungal pathogens such as *C. albicans* and *A. fumigatus* suggests that these organisms may bypass biosynthetic inhibition by importing exogenous folates. Notably, these transporters can utilize physiologically relevant folate forms, including methyl THF, the predominant circulating folate in humans [5]. Thus, effective antifungal strategies will need to target and inhibit both folate biosynthesis and transport functions. Since human cells lack both processes, this may be possible.

Eight of the nine *Arabidopsis thaliana* OPTs were evaluated, and only three showed an ability to transport folate. Among the nine *A. thaliana* OPT transporters, AtOPT6 has been demonstrated to transport GSH with relatively low affinity [37,38]. AtOPT4 has mainly been associated with oligopeptide transportbut is also shown to be capable of low-affinity GSH transport [39,40].

Identification of plant plasma membrane folate transporters could have important significance in folate biofortification strategies. Folate deficiency remains a major global health concern, particularly in pregnant women [41,42], and since commonly consumed staple foods such as rice, wheat, and potato provide insufficient folate [43–45], the identification of these transporters could add a new dimension to efforts at metabolic engineering of plants for folate fortification. This is particularly important considering the complex regulation of folate biosynthesis in plants. Metabolic engineering approaches in plants have successfully resulted in increased folate levels and, concomitantly, elevated PABA and pteridine levels, the toxicity of the latter being unknown [46,47].

## Specificity and mechanism of folate transport at the plasma membrane: insights from ScOPT1

The Opt1p of *S. cerevisiae* represents the best-characterized member of the OPT family. This transporter, which has been known for a long time as the sole GSH transporter, has been extensively characterized in terms of GSH transport. This detailed characterization has facilitated the understanding of this transporter and the mechanism of transport of not only GSH but also folate.

Opt1p is a 799-amino acid protein with 13 transmembrane domains (TMDs). Much of the initial analysis with the transporter assumed 12 TMDs, but later and more rigorous analysis using newer TMD prediction algorithms [48] suggested 13 TMDs. This was experimentally verified, and it was found that the N-terminal is cytoplasmic while the C-terminal is extracellular [48].

The protein has 12 cysteine residues and to identify the substrate-interacting residues investigations in this protein initial attempts were made to carry out the substituted-cysteine accessibility method. However, the cysteine-free protein was not functional, and attempts to identify functional revertants always led to two of the cysteines reverting back simultaneously (C622 and C632), suggesting their role in forming a disulfide bond [49]. These constraints prevented the use of this approach. As an alternate strategy to identifying the residues in the transporter involved in substrate recognition (and translocation), charged residues within the TMDs were mutated and evaluated [48,50,51]. Subsequently, all the TMDs were mutated residue by residue to evaluate each and every residue of the TMDs [22]. This alanine-scanning mutagenesis across all 13 TMDs identified residues important for substrate binding and translocation. All the mutations affecting activity fell within the predicted TMDs of the pore-lining helices of the *ab initio* model [48,52]. After the release of the AlphaFold2 structure, the *ab initio* model was modified to include only the loop regions predicted by AlphaFold2 because the AlphaFold2-predicted structure itself had much lower concordance with the experimental data and hence was not used [22]. These studies were carried out with GSH as the substrate. Kinetic evaluation of some of the mutants revealed a significantly higher *K*_m_ in some (V185A, Y374A, L429A, and Q526A), suggesting that these residues are involved with substrate binding.

When the same mutant data set was evaluated for folate transport, where again a sensitive growth assay was performed to evaluate functionality, it was interesting to note that most of the mutants that were defective for GSH transport were also defective for folate transport [22]. This would be expected for mutants involved in the translocation process. However, there were some mutants that were uniquely defective for GSH transport and others that were uniquely defective for folate transport (Table 3 and Figure 2). It is interesting that three of the four residues that were uniquely specific to GSH were in fact those that had a higher *K*_m_ in the kinetic analysis, further underlining their role in substrate binding.

**Table 3:** Residues specifically critical for either GSH or folate uptake in Opt1p

Residues important only for GSH uptake		Residues important only for folate uptake	
Residues	TMD	Residues	TMD
**V185**	TMD3	L231	TMD4
**G230**	TMD4	Y427	TMD7
**Y374**	TMD6	L428	TMD7
**Q526**	TMD9	L434	TMD7

**Figure 2:**
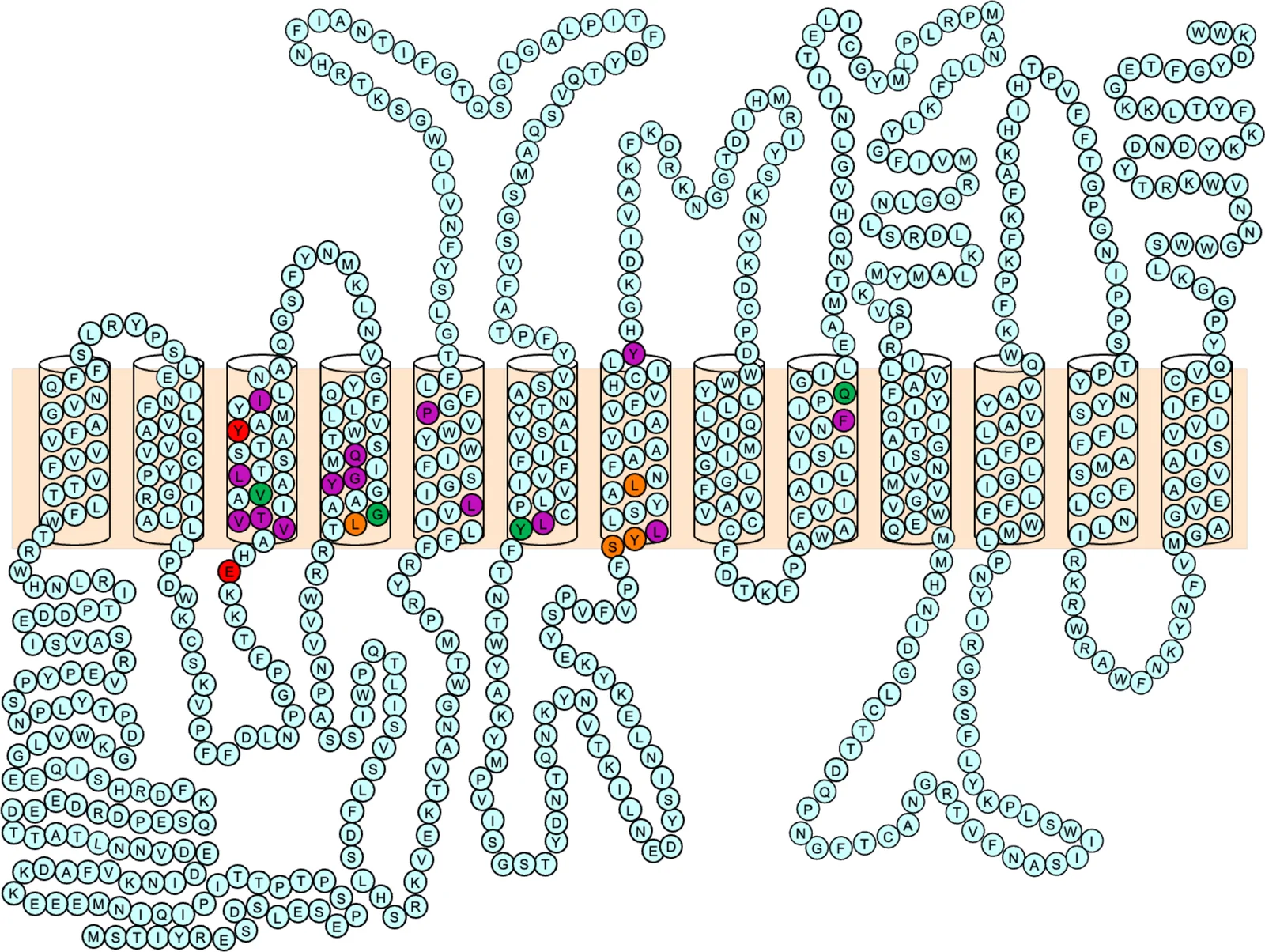
Topology of Opt1p The full-length OPT1p (799 amino acids) is depicted with 13 transmembrane helices, an extracellular N-terminal region, and a cytosolic C-terminal tail. Residues directly important for folate binding are highlighted in orange, those specific for GSH binding are shown in green, residues involved in both folate and GSH binding are highlighted in purple, and residues involved in proton-mediated transport are indicated in red. TM boundaries were based on [48].

In addition to folate and GSH, Opt1p has also been reported to transport a few oligopeptides (though with lesser affinity) and also mediate the uptake of extracellular pantetheine and 4′-phosphopantetheine after their conversion into mixed disulfides with GSH, suggesting that perhaps GSH would be the moiety recognized by the transporter [32,53,54]. Another report has also suggested that *S. cerevisiae* Opt1p is also a transporter of cysteine [55]. However, these latter findings were drawn solely on the basis of growth experiments, without recourse to confirmation with uptake experiments. While attempting to replicate these experiments in the same *S. cerevisiae gsh1Δ met15Δ* strain (Gupta and Bachhawat, unpublished observations), we observed that the conclusions drawn are artifactual, being a result of cross-feeding and the ability of neighboring cells to supply the minimal GSH requirements needed for growth, and that not only cysteine but also methionine can be observed to cause this phenotype, thereby discounting any role of Opt1p in cysteine uptake. Consistently, *OPT1* expression did not complement the growth defect of strains lacking the high-affinity cysteine transporter (*yct1Δ*) at low levels of cysteine (Gupta and Bachhawat, unpublished observations).

The evolutionarily most important substrate of a transporter that is transporting multiple substrates is best understood from its regulation and from the affinity for the different substrates. *OPT1* expression is strongly induced during sulfur starvation [56]. Cysteine and methionine strongly repress its expression while GSH exerts partial repression [57]. Thus, the overriding regulation is sulfur sources. There are secondary pathways regulating *OPT1* that include the Ssy1-Ptr3-Ssy5 amino acid-sensing pathway [58]. However, amino acids can enhance *OPT1* expression, with maximal induction only under sulfur-limiting conditions, underlining the key role of sulfur. The affinity of the transporter for GSH was also almost 10-fold higher than that for peptides [53], further underlining that its primary role is in GSH transport. The affinity for folate also appeared high (in growth assays), but the exact affinity could not be determined. More studies will have to be done to understand whether, under sulfur limitation, folate also has a role in the expression of the transporter.

Transport of GSH and folate by Opt1p is driven by a proton motive force [39,52]. Based on the dynamics of other proton-driven transporters that are well studied, the dynamics of the process should involve the initial binding of a proton to one of the residues, followed by the transfer of the proton to other residues in a relay process leading to the eventual translocation of the proton into the cell. This is coupled with the substrate binding and translocation. Two residues, E177 and Y193 in TMD3, which were also highly conserved, are likely essential for proton coupling [52]. Mutations of these residues were completely defective in both folate and GSH uptake, suggesting that both folate and GSH transport rely on a shared proton-coupled mechanism [33,52].

The mammalian proton-coupled folate transporter (SLC46A1), which functions optimally at acidic pH, has been investigated and shown to transport folate according to a detailed alternating-access model [59,60]. Opt1p also functions optimally at acidic pH. By analogy to the PCFT mechanism, therefore, a mechanism can be proposed [39]. In this mechanism, an initial protonation of E177, particularly at acidic extracellular pH, likely primes Opt1p into an outward-facing conformation. Subsequent substrate binding promotes gate closure and triggers a conformational shift to an inward-facing state, including a possible proton relay to Y193 that enables the coordinated release of the substrate and protons into the cytoplasm. Deprotonation then resets the transporter to its outward-facing state, completing the transport cycle. Thus, Opt1p represents a dual-specificity proton symporter in which conserved proton-coupling residues drive transport, while distinct lining residues determine whether folate or GSH is preferentially accommodated.

Recently, a transporter from barley, HvYS1, a YSL-clade member of the OPT family that is a phytosiderophore transporter, has been solved by CryoEM [61], and a mechanism of transport was also proposed. In the predicted model of transport of iron-mugeneic acid by HvYS1, the authors also suggested that the transport by the dimeric protein was occurring by an alternating access model. However, as HvYS1 had a fixed core distinct from the substrate binding site, the authors propose an alternating-access model that follows the elevator model [62] rather than a rocker switch model. The mutagenic data of Opt1p in contrast seems to suggest a rocker switch model, since in the elevator model the substrate-binding is confined, most often, to a single TMD. In the case of Opt1p, however, alanine scanning mutagenesis revealed that the residues that exclusively affect GSH transport (and are thus expected to be involved in GSH binding) fall in TMD3, TMD4, TMD6, and TMD9, while for folates they fall in TMD4 and TMD7. Thus, the substrate binding pockets span multiple TMDs. This seems to argue against an elevator model and in favor of a rocker switch model (Figure 3). HvYS1 has less than 20% identity with Opt1p, so eventually, the structure of Opt1p or a member of the PT-clade might be required to resolve this issue more conclusively.

**Figure 3:**
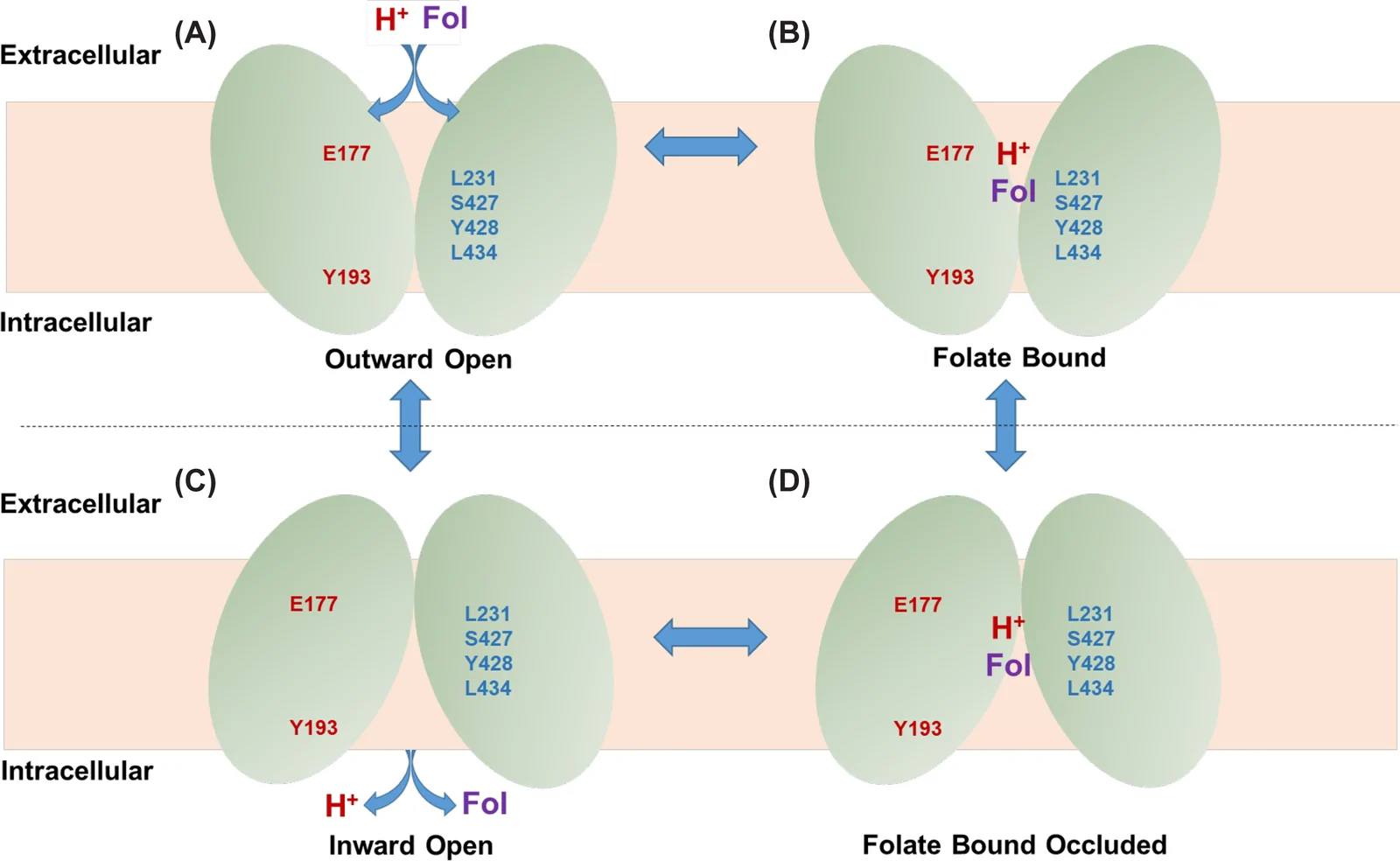
Proposed mechanism of proton-coupled transport of folate by Opt1p Based on functional and mutational analyses, Opt1p is proposed to function through this alternating-access mechanism driven by the proton motive force. (**A**) Under acidic extracellular conditions, protonation of key residues, particularly E177 and Y193, promotes an outward-open conformation that enables substrate binding. (**B**) Folate binds to the outward-open Opt1p transporter. (**C**) Binding folate induces a transition to an occluded state with the substrate trapped within the transporter. (**D**) Conformational rearrangement generates an inward-open state, allowing coordinated release of the substrate and protons into the cytoplasm. The empty transporter resets to the outward-open conformation for another transport cycle. Residues implicated in folate recognition include L231, Y427, L428, and L434. Conserved residues E177 and Y193 are proposed to mediate proton coupling required for transport of the substrates.

## Perspectives

- A family of folate transporters at the plasma membrane of fungi and plants have been identified that transport different folate forms into the cell and can supplement or even substitute the biosynthesis of folates in these organisms. An alternating access model stating details of proton symport is presented.
- Alleviating folate deficiency in humans is a worldwide problem, and supplementation with synthetic folates has met with some, but limited, success. Folate biofortification by metabolic engineering of crop plants seems still the best option, despite difficulties as a result of the complex biosynthesis of folate. These plasma membrane transporters could assist in the strategy.
- Manipulating plasma membrane folate transporter expression in plants along with external synthetic folate supplementation (foliar or in the soil) could enable uptake of folates and their subsequent conversion to high levels of natural folates. This could be an additional strategy in the basket of metabolic engineering approaches to plant folate biofortification.

## Open Access

Open access for this article is provided via our Subscribe to Open programme, made possible through the support of our subscribing institutions. Learn more here: https://portlandpress.com/pages/open_access_options_and_prices#conditional.

## Abbreviations

DHF: dihydrofolate
GSH: glutathione
OPT: oligopeptide transporter
PABA: *p*-aminobenzoic acid
THF: tetrahydrofolate
TMD: transmembrane domain
